# Bibliometric analysis and evaluation of the journal Medicina Oral Patología Oral y Cirugía Bucal (2008-2018)

**DOI:** 10.4317/medoral.23289

**Published:** 2020-01-01

**Authors:** Pilar Valderrama, Ángel Valderrama, Pilar Baca

**Affiliations:** 1 MsC, Scientific Information and Communication; 2 MsC, Digital Marketing and Communication in Social network; 3 PhD, Professor of Preventive and Community Dentistry. School of Dentistry. University of Granada

## Abstract

**Background:**

In 2008 the journal Medicina Oral Patología Oral y Cirugía Bucal was included in Journal Citation Reports. To appraise its evolution and current status, this study carried out a bibliometric analysis and evaluation of the journal for the period 2008-2018.

**Material and Methods:**

From the Web of Science, Journal Citation Reports we obtained the indicators Journal Impact Factor (JIF), 5-year JIF, JIF without self-cites, Eigenfactor score and Article Influence score (2010-2017); and from the Core Collection database the following variables: number and article types, institutions and countries of origin of the authors (2008-2018), and the variable cited and citing journal data in 2017. Twelve articles/year (n=132) were randomly selected to gather: the time between submittal and acceptance of an article, number of authors/article, representation of each section, gender of first author, and funding.

**Results:**

The journal occupied the third quartile of the JCR from 2010 to 2017, when it moved up to the second quartile. From 2008 to 2018 it published a total of 1,518 documents, 90% articles and 9.5% reviews. Sixty countries were represented, 48.68% of the documents coming from Spain, and overall 1,293 institutions were involved. Between submittal and acceptance of articles, the average time was 134.42 days, without differences between years. The mean of authors/article was 5.15, increasing over time. The sections most represented were Oral Medicine and Pathology, and Oral Surgery. There were no differences regarding the gender of the first author, and in general the authors did not provide information about funding received.

**Conclusions:**

The bibliometric results indicate a steadily improving position of this journal, along with a tendency to reduce self-citation. The time between reception of an article and its acceptance was very stable, the number of authors per article showed an increase, and there was a nearly equal representation of males and females as the first author.

** Key words:**Bibliometrics, Journal Impact Factor, Web of Science, gender.

## Introduction

In the year 1996, Medicina Oral S.L., a private enterprise created by Prof. José V. Bagán, founded the journal Medicina Oral with a first volume published in Sept.-Oct. of that year. This journal, with five issues per year, has always had international coverage. In addition to being the official publication of Spain´s Sociedad Española de Medicina Oral, it also represented the Academia Iberoamericana de Patología y Medicina Bucal (1). Indexed since its inception in Dialnet, its contents include Oral Medicine, Oral Pathology and Oral Surgery. The original language was Spanish, though after volume 2 number 4 (Aug.-Oct. 1997) all articles were published full in both languages, English and Spanish.

Medicina Oral was indexed in Index Medicus, MEDLINE and PubMed in both languages from volume 6 number 1 (Jan.-Feb. 2001) until its disappearance, the last issue being volume 9 number 4, in Aug.-Oct. 2004; and it was indexed in Scopus, Embase and Emcare from 1999 to 2004. It was also included in the Índice Médico Español and in the Índice Bibliográfico Español de Ciencias de la Salud, IBECS, during the period 2000-2004.

A new era began for the journal in 2004, when it changed its name to Medicina Oral, Patología Oral y Cirugía Bucal, to better reflect its main contents. Published in English, the first issue to come out with this name was volume 9 number 5, in Nov.-Dec. 2004 (2). Since that date, it has published six volumes per year, and the abbreviated title is Med Oral Patol Oral Cir Bucal.

At present, it is the official publication of one Iberoamerican and six Spanish professional societies. In addition to the two already mentioned, it represents the Sociedad Española de Odontología para el Minusválido y Pacientes Especiales, Sociedad Española de Cirugía Bucal, Sociedad Española de Gerodontología, Sociedad Española de Láser Odontoestomatológico and the Sociedad Española de Disfunción Craneomandibular y Dolor Orofacial.

The current sections and contents are:

1. Oral Medicine and Pathology. Clinicopathological as well as medical or surgical management aspects of diseases affecting oral mucosa, salivary glands, maxillary bones, as well as orofacial neurological disorders, and systemic conditions with an impact on the oral cavity; 2. Oral Surgery. Surgical management aspects of diseases affecting oral mucosa, salivary glands, maxillary bones, teeth, implants, oral surgical procedures. Surgical management of diseases affecting head and neck areas; 3. Medically compromised patients in Dentistry. Articles discussing medical problems in Odontology will also be included, with a special focus on the clinico-odontological management of medically compromised patients, and considerations regarding high-risk or disabled patients; 4. Implantology and 5. Periodontology.

The journal continues in this stage, to date, included in the same databases. On the 14th of May, 2009, both Medicina Oral and the current journal were evaluated and included in LATINDEX.

At that time the journal marked a new editorial line, coming out with a digital version (2) and establishing a process of on-line submittal to speed up the review of manuscripts. Peer-review had begun in the previous stage, and was now adopted systematically for all articles submitted. The editorial process strictly complied with the publication deadlines of each issue. In addition, the journal became more international, having incorporated prestigious people from various countries in its editorial committee. All these pre-requisites being met, and after careful evaluation, a letter was sent on the 25th of September, 2008, stating that Med Oral Patol Oral Cir Bucal was accepted for indexing in the Science Citation Index Expanded, SciSearch, and Journal Citation Reports (JCR) in Science Edition, beginning with volume 13, number 1 in 2008. Since 2010, based on the citations received by the articles published in the two previous years, it appears on the lists of journals included in the JCR with an impact factor of 1.071, situating it in the third quartile (Q3) of the only category to which it belongs: Dentistry, Oral Surgery and Medicine. In 2012 it was included in the PMC of the US National Library of Medicine, National Institutes of Health, USA (3).

During all its history, the journal has been directed by its founder, and for some years Professor Crispian Scully was co-director of the journal, until his death. It is currently the only Spanish journal included in JCR in this category. The change of scene has meant greater international status, and the journal´s inclusion in the above databases has made it possible to know the position and influence that it has in the worldwide journal network.

Bibliometrics are important tools for the quantitative analysis of scientific research productivity based on the number of articles and citations in peer-reviewed international journals. Bibliometric indicators are useful to evaluate internal affairs of a given journal, establish its relative position, and serve as a basis for strategic editorial policies. The best metric known is the Journal Impact Factor (JIF) (4), described by Garfield, but there are also variations: 5 Year JIF, and the JIF without self-cites. Further indicators of interest measuring the relative importance of a journal for the scientific community are the Eigenfactor score and the Article influence score (5).

Ten years after its inclusion on the lists of journals having impact, it is worthwhile to evaluate, using journal metrics, the evolution of Med Oral Patol Oral Cir Bucal and its relative position during this stage, as well as to characterize the articles published during this period. This performance analysis and study of the development of the journal represent an added value for it.

- Objective

The objective was to carry out a study of the main bibliometric indicators of the journal Med Oral Patol Oral Cir Bucal within the area Dentistry ever since it was included on the JCR list of journals with impact. In addition, an analysis of the journal based on the Web of Science (WoS) was carried out for the period 2008-2018, to evaluate the type of articles published, the countries and institutions publishing most in the journal, and the cited and citing journal data. Finally, taking a sample of articles, the evaluation was completed by taking into account the variables: time elapsed between submission and acceptance of an article, number of authors per article, gender of the first author, and funding.

## Material and Methods

This is a descriptive study founded on data from the WoS (© 2019 Clarivate Analytics), from the sections JCR and Core collection database, as well as on the articles published. Thus the study comprises two phases: bibliometrics and analysis of the articles.

- Bibliometrics

The first part entails a description of the basic bibliometric indicators, as well of those indicating the relative position of a journal. For these variables, the period of study was 2010-2017 and the data are from JCR (6). At the time of study, the data were not yet available for 2008 and 2009. The indicators were:

1. Journal Impact Factor (4). It is the indicator that measures the frequency with which the “average article” in a given journal and given year would be cited. Its calculation follows the formula: citations from JCR year of items published in the previous two years, divided by the total number of ciTable items (articles and reviews).

2. 5 Year JIF: This indicator refers to the citations received by a journal, in one year, of the ciTable articles published in the five previous years. The calculation is factored in the same manner as the JIF, the difference being the five-year window of citation data.

3. JIF without Self-Cites: Similarly, an impact factor indicator, but featuring an exceptional difference. Any citation to a publication from the same journal is excluded when calculating the IF. Elsewise, its calculation is identical to that of the JIF. Hence, in view of this indicator and the JIF, the percentage of self-citation could be assessed through the following formula: (JIF-JIF without self-cites/JIF) x 100.

4. Eigenfactor Score (ES). Measures the full importance of a journal within the scientific community. The sum of the ES taking into account all JCR journals is 100. The ES is based on how many times articles from the journal published in the past five years have been cited in the JCR year, removing journal self-citation; but it also considers which journals have contributed these citations so that highly cited journals will influence the network more than lesser cited journals.

5. Article Influence Score (AIS). This index quantifies the average influence of a journal's articles over the first five years after publication. It is calculated by multiplying the ES by 0.01, and then dividing by 5 (years) the number of articles in the journal, normalized as a fraction of all articles in all publications.

- Analysis of the articles

The second part of this study evaluates and characterizes the contributions of the journal during the period 2008-2018. Some data were obtained from the WoS Core collection database (7). The variables selected were the number and type of document, and the countries and organizations that most heavily published in that journal. To approach the analysis of citations, the ones made in the journal (citing) and the ones received (cited) were accounted for, choosing the year 2017. Finally, to complete the characterization of the articles published by the journal in the same time period, 12 articles per year were selected randomly. The variables considered were: 1, time between reception of articles and their acceptance; 2, number of authors per article; 3, number of articles pertaining to the different sections of the journal; 4, gender of the first author and 5, funding received.

- Statistical analysis

To calculate the sample size of the set of selected articles, the variables taken as reference were: time of acceptance and number of authors. They were held to be more informative than the categories gender, type of article and subject classification. Considering a standard level of significance of α=0.05, a power of 80% and a standard statistical difference over deviation of 0.25, which is low according to the scale of Cohen [1988] (8), the final sampling size was 128. In order to get a uniform value over the years, this study focused on 12 articles in each of the 11 years, which means a total of 132.

In the statistical analysis, the Gaussian distribution of the variable representing the time elapsed between submittal and acceptance was contrasted in the different years of study by means of the Kolmogorov-Smirnov test, and after confirming the homogeneity of variances using the Levene test, ANOVA was applied to test the hypothesis of equality of means over the different years.

Regarding the number of authors per article, and per year, we considered whether there was a significant growing trend by testing the significance of the slope of the adjusted least-squares straight line.

The variables representing funding and the number of articles of the different sections of the journal were studied in terms of percentages over the total, and a test of multiple comparisons of proportions was formulated, to be resolved by means of the Chi-squared test. The gender of the first author was also analyzed percentagewise, comparing the percentages of men and women by means of the hypotheses test H0: *P*=0.5 versus H1: P≠0.5, resolving it again by means of the Chi-squared statistic.

## Results

- Bibliometric indexes of the journal

**Figure 1:**
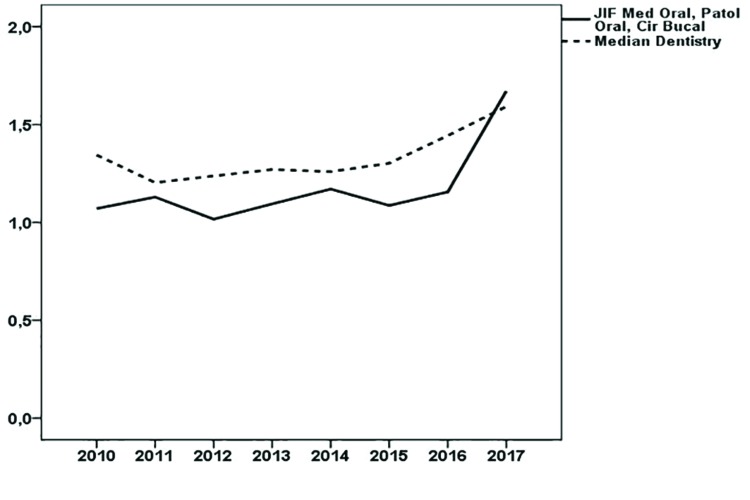
Evolution of the Impact Factor of the journal Med Oral Patol Oral Cir Bucal in the period 2010-2017. Comparison with the median for the category Dentistry, Oral Surgery and Medicine.

The values for the bibliometric indexes obtained by the journal are shown in Table 1, along with the quartile and percentile occupied according to the JIF. From 2010 to 2016 it was situated in Q3, to later move up to Q2 in the year 2017, where it occupied a JIF percentile of 55.5%. The 5 year JIF indicator shows an evolution parallel that of to the JIF. In contrast, the JIF without self-cites rises at an even greater pace. Fig. 1 displays the evolution of the JIF compared with the median of the category. Table 1 also shows the bibliometric indicators ES and AIS.

**Table 1:**
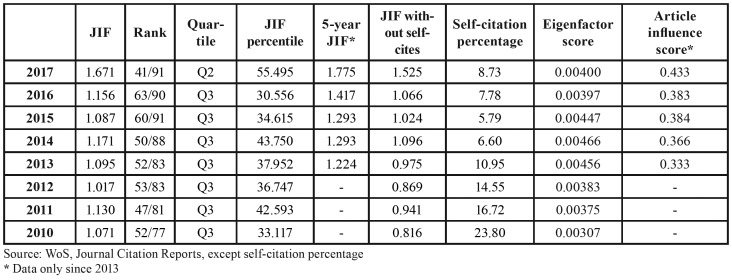
Med Oral Patol Oral Cir Bucal. Journal impact factor (JIF), and other bibliometrics based on the number of citations. Rank, quartile and percentile in the category Dentistry Oral Surgery and Medicine.

- Analysis of the articles

Table 2 shows, according to WoS data, by years and globally, the number and type of articles published in Med Oral Patol Oral Cir Oral during the period 2008-2018. When limited to original articles and reviews, the former amount to 90.46%, whereas the reviews represent just 9.53%.

The countries and organizations that have published most in this journal in the period 2008-2018 are indicated in Table 3. Authors from 70 countries published 1,518 documents; Spain represents 48.68%, followed by Brazil (15.55%) and Turkey (7.38%). The organizations most representative of the journal were Spain´s public universities, the University of Valencia occupying first place.

In 2017, the journal received 2,621 citations of articles published to date (including the year 2017). At the same time, the journal made a total of 3,069 citations to other journals in that year. The journals most cited by it and the journals that cite it the most are shown in Table 4.

**Figure 2:**
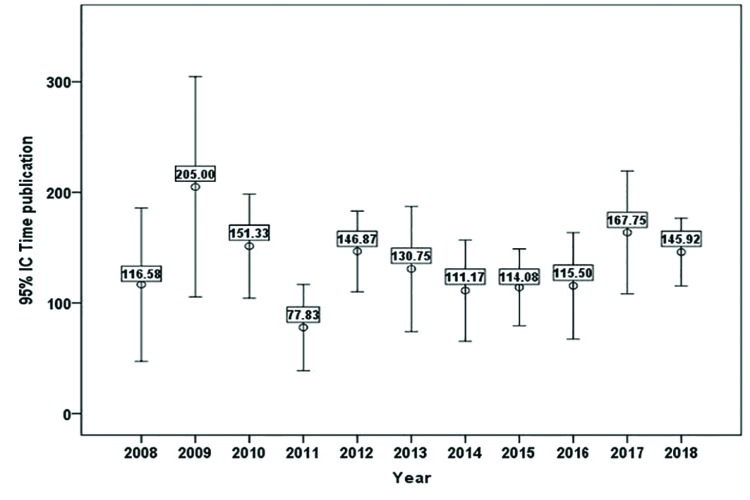
Mean time in days and 95% confidence intervals, between reception of an article and its acceptance in Med Oral Patol Oral Cir Bucal during the period 2008-2018.

In view of the articles selected at random, it was determined that the time lasted between the reception of a manuscript and its definitive acceptance was, on the average, 134.42 days (with a standard deviation of 88.47), the minimum being 8 days and the maximum 647. Fig. 2 offers a graphic display, in error bars, of the mean time as measured in years. Results of the ANOVA test indicate that there were no significant differences over the 11-year period analyzed.

**Figure 3:**
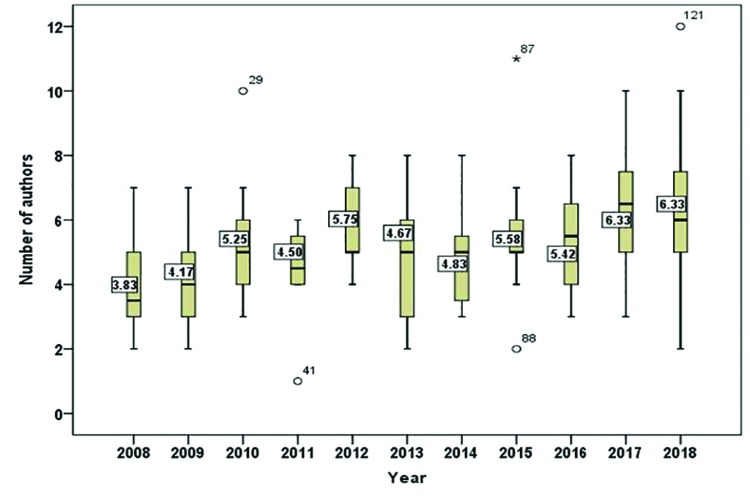
Box-Whiskers diagram of the number of authors per article each year in the journal Med Oral Patol Oral Cir Bucal during the period 2008-2018. Included is the mean value.

Globally, the mean number of authors per article was 5.15 (with a standard deviation of 1.904), the minimum being one author and the maximum 12. Fig. 3 represents, yearly, the number of authors per article including the mean. The mean number of authors per article underwent a significant linear growth over the period of study (*p*=0.001).

**Table 2:**
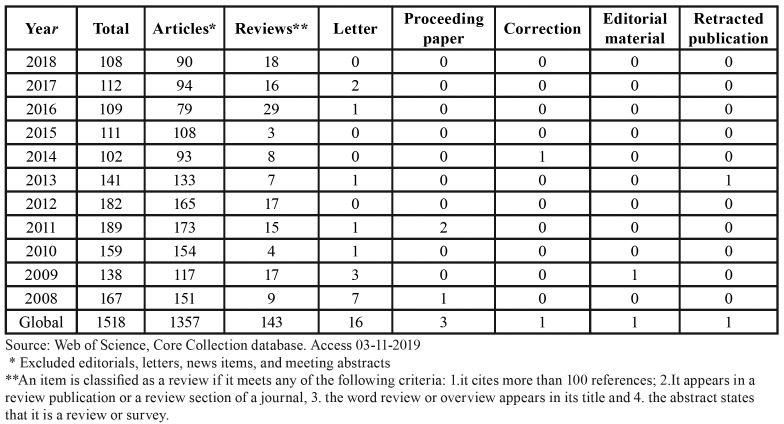
Number of articles, reviews and other document types from 2008 to 2018 in the journal Med Oral Patol Oral Cir Bucal.

**Table 3:**
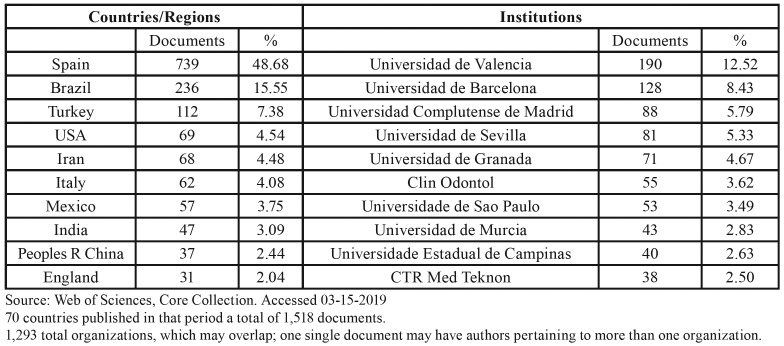
Countries and Institutions publishing most in the journal Med Oral Patol Oral Cir Bucal from 2008 to 2018.

**Table 4:**
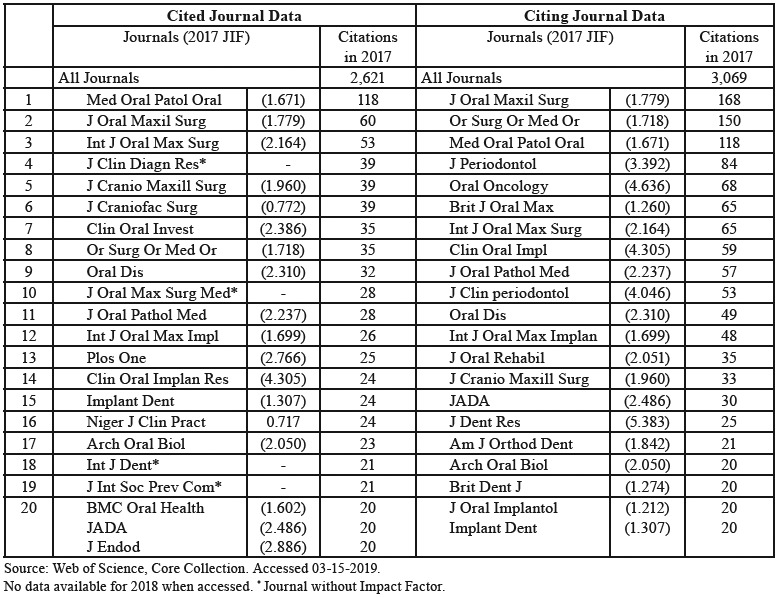
Journals most cited by and most citing Med Oral Patol Oral Cir Bucal Journal in 2017.

Considering the five main sections of the journal we found the following percentages of published articles: Oral Medicine and Pathology (43.18%), Oral Surgery (31.82%), Clinical and Experimental Dentistry (9.85%), Medically compromised patients in Dentistry (6.82%) and Biomaterials (3.79%). Analyzing the differences between percentages, it was concluded that there were not significant differences between the first two sections, nor among the latter three.

If we focus on the gender of the first author, there is a majority of males (72 versus 60 females), but the difference does not prove significant (*p*=0.5455).

The matter of funds received for carrying out research in the field was not mentioned by 79.55% of the articles; 19.70% stated they had used public funds, and just 0.76% acknowledged private funding, thus leading to a statistically significant difference (*p*<0.001).

## Discussion

In this study, a bibliometric analysis of the Med Oral Patol Oral Cir Bucal journal was carried out according to WoS; moreover, the objective was to analyze certain variables held to be of interest in the published articles.

The JIF was the main indicator of reference, but we also relied on the variants 5 year JIF and JIF without self-cites. Despite the controversy surrounding its usage and possible manipulation (9), JIF is still held up as the gold standard. It is not perfect, but it is a sound reference when it comes to evaluating the quality of individual publications (10).

Deserving mention here is the fact that, when in 2010 the journal joined the ranks of the JIF list, it went directly to Q3, and then kept its place in this quartile. In 2017 it achieved an important increase, surpassing the median for the field Dentistry Oral Surgery and Medicine, hence moving up to Q2 (Fig. 1). This improvement in the frequency of citation could be attributed, in part, to the fact that the number of reviews in recent years has increased (Table 2), and this type of document is cited more frequently (11). The relationship between the JIF and the JIF without self-cites evidences an interesting evolution that reflects improvement of the journal. In 2010, self-cites of the journal contributed to its JIF by 23.80%, yet they have gradually decreased to percentages below the global ones for the category (12). It is known that one of the problems attributed to the JIF is precisely the temptation to self-cite in order to move up in one´s JIF ranking (9).

Considering the number of documents, there has been a decline in recent years: a maximum value was reached in 2011 (n=189) and a minimum in 2018 (n=108). At present, given the worldwide growth in the number of published articles (13,14), this reduction would reflect an editorial policy aimed at making a careful selection, especially taking into account the fact that it entails no reduction of the JIF.

Regarding the type of documents, it is important to highlight the balance between original research and review articles. Reviews amounted to 9.5%, though over the past 5 years they have come to stand for 13.57%. This tendency to publish more reviews is a phenomenon affecting diverse fields of science, and Dentistry is surely no exception (15). It is known that some types of reviews –systematic and meta-analyses— are reaching epidemic proportions (16). Regardless of their unquestionable utility, this type of article tends to be cited more often (11). In Med Oral Patol Oral Cir Bucal, the original articles amount to just over 90%; and we should not forget that they are the foundation for advancement in scientific knowledge, the raw material of reviews. An excess of reviews could inflate the JIF, but it would eventually lead to an impoverishment of science, and of the journal in question.

It is interesting to note that it is exceptional that this journal publishes other types of documents —conferences, proceedings, letters, etc.— that could be cited and increase the numerator in the JIF calculation, but they are not taken into account when defining the denominator. That is, they would artificially favor a higher JIF (9).

The internationalization of this journal has been important. The authors of the 1,518 articles published in the period 2008-2018 came from 70 countries, and just over half (51.32%) were not from Spain. Although the authors were from all over the world, outstanding in number are the contributors from Brazil and Turkey, followed by the USA, Iran and Italy. Organizations also reflect author origin. The top 5 in the case of this journal represent Spain´s public universities, even though positions 7 and 9 are occupied by universities from Brazil.

For the analysis of the journals most cited by Med Oral Patol Oral Cir Bucal, as well as their sources of citation, the year 2017 was selected, this being the most recent year with available WoS at the time of study. The number of citations made is greater than those received (3,069 versus 2,621). A total of 23 journals (19 included in JIF) cited Med Oral Patol Oral Cir Bucal at least on 20 occasions, while 22 journals (all included in JIF) were cited by it, again on more than 20 occasions. These facts are relevant when analyzing bibliometric data such as the JIF, and to know the scientific market they share. There appears to be a certain reciprocity between journals and citations. Of the 20 most-cited and most-citing ones, we find 13 in common. For instance, except for the journal itself, the one most often citing the journal of study was J Oral Maxil Surg (n=60), which also happened to be the journal most cited by Med Oral Patol Oral Cir Bucal (n=168).

When the articles were selected randomly, one parameter that was available and appraised as useful for researchers was the average time elapsing between a manuscript´s reception and its acceptance. The most likely period was determined to be 4-5 months. Fig. 3 shows that this period has been quite sTable from 2008 to 2018, without significant differences, which would indicate steady and rigorous editorial policy, a true strongpoint for a journal. It is often the case that journals included in the JCR prolong the review of an article to an excessive extent.

The mean number of authors per article shows a significant upward trend, from 3.83 in 2008 to 6.33, eleven years later. This finding, observed largely in Medicine (17) and in Dentistry (18,19), could be explained by the need to increase academic productivity for personal promotion within the institutional ranks, but it also suggests a greater complexity or sophistication in research projects, and more collaborative efforts (20). The truth is that this growing tendency has originated debate, and has led the International Committee of Medical Journal Editors to establish well-defined and stricter criteria for authorship and contributorship (21), definitions which should be accepted as guidelines by the editorial teams of the journals.

Regarding the gender of the first author, although the percentage of men is higher (54.54%), there are no significant differences between gender in the journal, contrary to what is usual in both Medicine (22), and Dentistry (23,24), where male authors predominate. The first author position should be clearly assigned to the individual making the greatest contribution (25).

Also of interest is the fact that 78.79% of the articles do not provide any information about the funding of the research. Given this percentage, one suspects that it is an involuntary, not deliberate, omission of information. The policy of the journal should specifically emphasize the relevance of this information, since the research should be transparent. In fact, it has been shown that the financing of the articles is a factor that influences the JIF (26).

## Conclusions

The results of bibliometric indicators point to an ascending evolution of the journal Medicina Oral Patología Oral y Cirugía Bucal, situated in 2017 in Q2 within the category Dentistry, and a tendency for it to reduce self-citation. Since it joined the ranks of the JCR in 2008, the journal has put out 1,518 documents —nearly all articles and reviews— submitted by authors from 1,293 institutions and 70 countries. There are, apparently, “relationship journals” showing reciprocity in their citations. The journal has a sTable time period between submittal and acceptance of articles, a growing trend in the number of authors per article, and a nearly equal ratio of men and women as the first authors. When interpreting results, one must bear in mind that data came from the WoS database, without taking into account others, such as SCOPUS. The present study will help journal readers to better understand the current state and the evolution of Med Oral Patol Oral Cir Bucal.
